# An Interpretable Ensemble Transformer Framework for Breast Cancer Detection in Ultrasound Images

**DOI:** 10.3390/diagnostics16040622

**Published:** 2026-02-20

**Authors:** Riyadh M. Al-Tam, Aymen M. Al-Hejri, Fatma A. Hashim, Sachin M. Narangale, Mugahed A. Al-Antari, Sarah A. Alzakari

**Affiliations:** 1 Faculty of Administrative and Computer Sciences, University of Albaydha, Albaydha CV46+6X, Yemen; 2 School of Computational Sciences, Swami Ramanand Teerth Marathwada University, Nanded 431606, Maharashtra, India; 3 Faculty of Engineering, Helwan University, Cairo 11795, Egypt; 4 Applied Science Research Center, Applied Science Private University, Amman 11937, Jordan; 5 School of Media Studies, Swami Ramanand Teerth Marathwada University, Nanded 431606, Maharashtra, India; 6 Department of AI and Data Science, Sejong University, Seoul 05006, Republic of Korea; 7 Department of Computer Sciences, College of Computer and Information Sciences, Princess Nourah Bint Abdulrahman University, P.O. Box 84428, Riyadh 11671, Saudi Arabia

**Keywords:** breast cancer detection, ultrasound imaging, vision transformer, transfer learning, ensemble learning, computer-aided diagnosis (CAD)

## Abstract

**Background/Objectives**: Early and accurate detection of breast cancer is essential for reducing mortality and improving patient outcomes. However, the manual interpretation of breast ultrasound images is challenging due to image variability, noise, and inter-observer subjectivity. This study aims to address these limitations by developing an automated and interpretable computer-aided diagnosis (CAD) system. **Methods**: We propose an automated and interpretable computer-aided diagnosis (CAD) system that integrates ensemble transfer learning with Vision Transformer architectures. The system combines the Data-Efficient Image Transformer (Deit) and Vision Transformer (ViT) through concatenation-based feature fusion to exploit their complementary representations. Preprocessing, normalization, and targeted data augmentation enhance robustness, while Gradient-weighted Class Activation Mapping (Grad-CAM) provides visual explanations to support clinical interpretability. The proposed model is benchmarked against state-of-the-art CNNs (VGG16, ResNet50, DenseNet201) and Transformer models (ViT, DeiT, Swin, Beit) using the Breast Ultrasound Images (BUSI) dataset. **Results:** The ensemble achieved 96.92% accuracy and 97.10% AUC for binary classification, and 94.27% accuracy with 94.81% AUC for three-class classification. External validation on independent datasets demonstrated strong generalizability, with 87.76%/88.07% accuracy/AUC on BrEaST, 86.77%/85.90% on BUS-BRA, and 86.99%/86.99% on BUSI_WHU. Performance decreased for fine-grained BI-RADS classification—76.68%/84.59% accuracy/AUC on BUS-BRA and 68.75%/81.10% on BrEaST—reflecting the inherent complexity and subjectivity of clinical subclassification. **Conclusions**: The proposed Vision Transformer-based ensemble demonstrates high diagnostic accuracy, strong cross-dataset generalization, and clinically meaningful explainability. These findings highlight its potential as a reliable second-opinion CAD tool for breast cancer diagnosis, particularly in resource-limited clinical environments.

## 1. Introduction

Breast cancer remains a leading global health crisis, surpassing lung cancer in 2020 as the most commonly diagnosed malignancy, accounting for 11.7% of all new cancer cases [1,2]. In 2022, approximately 2.3 million new cases were reported globally, leading to an estimated 670,000 deaths [3]. Survival rates show a severe disparity, ranging from over 90% in high-income countries to as low as 40–50% in low-income nations [4]. This alarming trend underscores the urgent global need for accessible, accurate, and resource-appropriate diagnostic solutions, a goal championed by the WHO Global Breast Cancer Initiative (GBCI) [4].

Breast cancer development is linked to several modifiable (e.g., alcohol, obesity, smoking) and non-modifiable (e.g., genetic predisposition like BRCA1/2 mutations) risk factors [5,6,7]. Common clinical signs include palpable lumps, skin dimpling, and abnormal discharge [8]. Medical imaging is critical for early detection. Key modalities include Mammography [9,10,11], Magnetic Resonance Imaging (MRI) [12,13], and Ultrasound [14]. Breast Ultrasound is particularly valuable for evaluating dense breast tissue and differentiating between solid masses and fluid-filled cysts without using ionizing radiation [6,15]. A significant advancement is Automated Breast Ultrasound (ABUS), which provides standardized, comprehensive, and consistent imaging of the entire breast, reducing operator dependency compared to conventional handheld methods [16,17].

**Figure 1:**
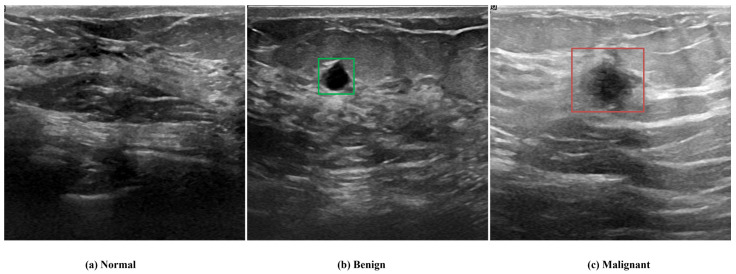
Representative ultrasound images from the BUSI dataset: (**a**) normal breast tissue, (**b**) benign lesion (outlined in green), and (**c**) malignant lesion (outlined in red).

Despite these technologies, radiologists still face challenges in interpreting images due to variations in imaging features, inconsistencies between ultrasound devices, and the subtle visual differences between normal tissue, benign lesions, and malignant tumors, as presented in Figure 1. Therefore, several deep learning studies have been conducted with the aim of improving the early detection and classification of breast cancer and supporting radiologists in enhancing diagnostic accuracy and streamlining workflow efficiency [18,19,20,21,22,23].

Building on these advances, this study proposes a novel CAD framework that integrates pre-trained convolutional neural networks (CNNs) and Vision Transformers (ViTs) into ensemble models. We evaluate a range of CNN architectures (VGG16, VGG19, MobileNetV2, ResNet50, Xception, InceptionV3, InceptionResNetV2, and DenseNet201) and transformer variants (ViT, Deit, Dit, Swin, Beit, ViT-Hybrid), both individually and in ensemble configurations, including a novel Deit + ViT ensemble. This approach aims to harness complementary feature representations to enhance classification performance across multiple breast ultrasound categories.

The remainder of this paper is organized as follows: Section 2 reviews related work on AI-based breast cancer detection, Section 3 describes the proposed methodology, Section 4 presents experimental results, Section 5 discusses the findings in comparison with prior studies, and Section 6 concludes the study.

## 2. Related Works

Numerous studies have explored deep learning (DL) and machine learning (ML) techniques for breast ultrasound (BUS) image classification, yielding significant advances in automated diagnosis. These approaches can be broadly categorized into traditional CNN-based models, ensemble and transfer learning techniques, and real-time clinical application systems.

### 2.1. Traditional Deep CNN Architectures for BUS Classification

Several studies have employed standard convolutional neural network (CNN) architectures to classify BUS images. A multistage transfer learning approach, for instance, fine-tuned pre-trained models such as VGG-16 and ResNet-50 on BUS datasets for effective classification.

Alotaibi et al. (2023) [24] introduced a three-step image preprocessing pipeline—speckle noise filtering, ROI highlighting, and RGB fusion—to enhance ultrasound image quality for breast tumor classification. Applied to VGG19 using transfer learning across BUSI, KAIMRC (5693 images), and Dataset B (162 images), the preprocessing improved recall from 76.8% to 87.4% and F1-score from 75.8% to 87.4%, with the best model achieving 87.8% accuracy on the BUSI dataset.

AlZoubi et al. [25] conducted a comparative evaluation of six transfer learning-based deep CNN models and an automatically designed CNN (BONet) using a dataset of 3034 2D ultrasound images. BONet, optimized via Bayesian methods, outperformed other models, achieving 83.33% accuracy, a low generalization gap (1.85%), and reduced model complexity (~0.5 M parameters). The study also employed saliency maps to enhance interpretability, demonstrating BONet’s potential clinical applicability.

Altameemi et al. [26] proposed the Deep Neural Breast Cancer Detection (DNBCD) model, an explainable deep learning framework for classifying breast cancer using histopathological and ultrasound images. Built on DenseNet121 with custom CNN layers and Grad-CAM for interpretability, the model was evaluated on BreakHis-400× and BUSI datasets, achieving accuracies of 93.97% and 89.87%, respectively. The study emphasizes model transparency and clinical applicability, outperforming several existing methods.

In a large-scale study, the authors of [27] suggested a VGG-based CNN trained on 14,043 ultrasound images gathered from 32 hospitals in a comprehensive investigation. The model performed on par with experienced radiologists, with an accuracy of 86.4% and an AUC of 91.3%. Another work [28] introduced a fully automated, multi-stage pipeline that combined lesion segmentation and classification. By evaluating various CNN architectures and using ensemble strategies, it achieved a Dice coefficient of 82% and a classification accuracy of 91%. A cyclic mutual optimization mechanism allowed for iterative refinement between segmentation and classification, boosting diagnostic performance.

Further comparative studies, such as [29], assessed models including InceptionV3, VGG16, ResNet50, and VGG19 on a dataset of 5000 training and 1007 test images. InceptionV3 achieved the highest accuracy of 82.8% and AUC of 90.5%. Similarly, Liao et al. [30] evaluated VGG19, ResNet50, DenseNet121, and InceptionV3 on a smaller dataset of 256 images, with VGG19 achieving an AUC of 98% and an accuracy of 92.95%.

These findings underscore the effectiveness of traditional CNNs for BUS classification, especially when combined with interpretability tools, ensemble enhancements, and optimization strategies.

### 2.2. Ensemble and Transfer Learning Approaches

To address the limitations of single-model architectures, many studies have employed ensemble methods and transfer learning to improve classification performance.

In this context, Zhou et al. [31] investigated Vision Transformers (ViT) for BUS classification and demonstrated that ViTs outperformed traditional CNNs, especially when self-supervised learning was used. An ensemble of ten independently trained ViTs achieved an impressive AuROC of 0.977, AuPRC of 0.965, and classification accuracy of 93.8% on benign and malignant cases from the BUSI dataset. Islam et al. [32] introduced an Ensemble Deep Convolutional Neural Network (EDCNN) that combines the MobileNet and Xception architectures. Their model incorporated various preprocessing steps, such as normalization and data augmentation, and achieved an accuracy of 87.82% and an AUC of 91% on the BUSI dataset. The integration of Grad-CAM further enhanced the model’s interpretability.

Furthermore, a deep learning-based pipeline for discriminating between benign and malignant lesions was proposed [33], using a biopsy-confirmed dataset of 2058 BUS masses. Transfer learning models—InceptionV3, ResNet50, and Xception—outperformed a shallow CNN (CNN3) and traditional ML models with handcrafted features. Among them, InceptionV3 yielded the best standalone results with 85.13% accuracy and an AUC of 91%. Notably, fusing deep features from all three models further improved accuracy to 89.44% and AUC to 93%, underscoring the effectiveness of feature-level fusion. Another study [34] trained a generic deep learning model on ultrasound data from 82 malignant and 550 benign cases, achieving an AUC of 84% and specificity of 80.3%. Similarly, a comparative study [35] evaluated traditional ML, CNNs, and Google AutoML Vision using the BUSI and Mendeley BUS datasets. AutoML achieved 86% accuracy and an F1-score of 83%, demonstrating the promise of automated architecture search.

Generally, studies have limitations such as class imbalance, and the absence of external validation restricts their generalizability. Additionally, the lack of preprocessing and dedicated segmentation steps may have affected its diagnostic robustness.

### 2.3. Hybrid and Multi-Task Architectures

Recent studies have explored hybrid and multi-task learning (MTL) approaches to enhance the diagnostic capabilities of BUS classification systems. These methods aim to leverage the strengths of multiple network types or tasks simultaneously—such as segmentation and classification—to improve overall performance and clinical relevance. In this context, Ejiyi et al. [36] proposed SegmentNet, a hybrid CNN architecture that integrates Distance-Aware Mechanisms (DaMs) and Local Feature Extractor Blocks (LFEBs). This design allowed the model to effectively capture both global context and fine-grained local information. SegmentNet achieved a segmentation accuracy of 93.88% on the BUSI dataset, highlighting the benefit of spatially aware architectural components in delineating lesion boundaries.

In another hybrid approach, a combination of AlexNet, ResNet, and MobileNetV2 was used to create a deep ensemble model that incorporated residual learning and depth-wise separable convolutions [37]. This model demonstrated impressive results, achieving 96.92% accuracy in abnormality detection and 94.62% in malignancy classification on the BUSI dataset. The fusion of architectures contributed to both feature diversity and computational efficiency.

In line with the growing emphasis on multimodal learning, one study developed and compared breast cancer classification models based on both mammography and ultrasound images against their single-modal counterparts [38]. Utilizing imaging data from 790 patients—comprising 2235 mammograms and 1348 ultrasound scans—the researchers evaluated six deep learning models (ResNet-18, ResNet-50, ResNeXt-50, Inception v3, VGG16, and GoogleNet) using standard metrics such as AUC, sensitivity, specificity, and accuracy. The multimodal model achieved superior results in specificity (96.41%), accuracy (93.78%), precision (83.66%), and AUC (0.968) when the ResNet-18 model was used as a baseline. Heatmap visualization was employed to validate the multimodal model’s decision-making process. These findings underscore the diagnostic benefits of fusing complementary imaging modalities, which may enhance early breast cancer detection and decision support in clinical settings.

Multi-task learning frameworks have also gained traction for their ability to simultaneously address classification and segmentation tasks. One such study proposed an end-to-end system combining nU-Net and UNet++ to classify breast lesions into benign, malignant, and normal categories while concurrently performing lesion segmentation [39]. The model achieved an accuracy of 80.20% on the BUSI dataset, demonstrating the potential of task synergy to enhance diagnostic performance, particularly in limited-data scenarios.

Overall, hybrid and multi-task architectures represent a promising direction in BUS classification research, combining spatial, contextual, and task-level learning to address the limitations of single-purpose models. However, these models often demand increased computational resources and require careful tuning to balance multiple objectives effectively.

### 2.4. Real-Time and Clinical Workflow-Oriented Applications

Even though BUS classification models work well in experiments, their clinical application is still not fully explored. An AI-based CAD system was assessed in a sequential clinical workflow in a real-world study carried out in a Korean hospital [40]. The system’s single-institution character hindered generalizability, although it increased diagnostic performance (AUC of 85.5%, accuracy of 85.4%). A 3D-DCNN model with a unique threshold loss for automated breast ultrasound (ABUS) was created in a similar setting [41]. Its sensitivity on a 614-volume dataset was 95%. In another study, 1600 BUS images were used to test a fully automated detection model that combined DenseNet and U-Net [42], achieving an accuracy of 96% and AUC of 99%. Finally, deep learning-based data fusion techniques are increasingly being explored for integrating heterogeneous cancer data sources to improve diagnostic accuracy and interpretability [43]. These methods hold promise for enriching CAD systems by leveraging multi-source information, including imaging, pathology, and clinical data.

In conclusion, ML and DL techniques for BUS classification have achieved promising results across diverse datasets and model architectures. However, several limitations persist. Many studies rely on small or institution-specific datasets, hindering generalizability and introducing bias when compared to those using public benchmarks. The absence of external validation often limits assessments of model robustness. Additionally, crucial clinical information—such as BI-RADS scores, lesion size, and patient demographics—is rarely incorporated, reducing clinical relevance. Real-time applicability is also frequently overlooked.

## 3. Materials and Methods

Ultrasound imaging, or sonography, plays a critical role in the detection and diagnosis of breast cancer due to its safety, affordability, and effectiveness. However, interpreting breast ultrasound (BUS) images can be challenging, often requiring expert radiological assessment. To support clinical decision-making, we propose a Computer-Aided Diagnosis (CAD) system that leverages state-of-the-art AI models for the accurate and reliable classification of BUS images. The system addresses three key classification tasks: (1) distinguishing between normal, benign, and malignant categories; (2) binary classification of benign versus malignant lesions; and (3) prediction of BI-RADS categories to enhance clinical risk stratification.

**Figure 2:**
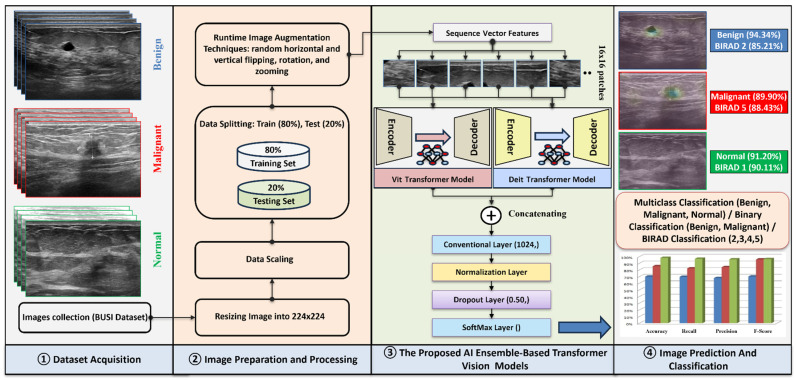
The proposed ensemble-based Vision Transformer model for breast cancer classification using ultrasound images.

As illustrated in Figure 2, the proposed methodology involves several critical stages, beginning with data preparation and preprocessing—including image resizing, scaling, dataset splitting, and data augmentation—to ensure model robustness and generalizability. We evaluate a broad spectrum of state-of-the-art convolutional neural network (CNN) architectures, including VGG16 [44], VGG19 [45], ResNet50 [46], DenseNet201 [47], MobileNetV2 [37], Xception [48], InceptionResNetV2 [49], and InceptionV3 [50], all of which have demonstrated strong performance in medical image analysis.

In addition to CNNs, we assess the performance of advanced transformer-based vision models, such as Deit [51], Dit [52], Beit [53], Swin [54], ViT-Hybrid [55], and ViT [23], to explore their applicability to BUS classification. All models utilize transfer learning by initializing from ImageNet-pretrained weights and fine-tuning on the target dataset to leverage learned representations.

To enhance classification accuracy and robustness, we implement ensemble strategies that integrate predictions from two or more models using a feature-level concatenation layer [23]. Finally, we apply Gradient-weighted Class Activation Mapping (Grad-CAM) to interpret and visualize the decision-making process of the ensemble models, providing insight into the regions of interest that influenced the predictions.

### 3.1. Data Acquisition

This study utilizes the publicly available Breast Ultrasound Dataset (BUSI) (Al-Dhabyani et al., Assiut University Hospital, Assiut, Egypt) [56], which comprises breast ultrasound images categorized into three classes: normal, benign, and malignant. The dataset was collected in 2018 from 600 female patients, aged between 25 and 75 years. It includes a total of 780 ultrasound images in PNG format, each with an average resolution of 500 × 500 pixels. The distribution of images across the three categories is as follows: 133 normal, 437 benign, and 210 malignant cases. This class imbalance reflects real-world clinical scenarios and is addressed during the data preprocessing phase.

### 3.2. Data Preparation and Preprocessing

Preprocessing is a critical step to ensure the dataset is clean, consistent, and suitable for training deep learning models. The original dataset contained approximately 1100 ultrasound images; however, following preprocessing steps guided by Baheya radiologists, the dataset was refined to 780 images [56]. This reduction involved removing duplicate images and correcting mislabeled annotations to ensure data quality and integrity. The original images, stored in DICOM format, were converted to PNG using Medixant RadiAnt DICOM Viewer, (2025.2) facilitating compatibility with image processing pipelines. Each image was then categorized into one of three classes: normal, benign, or malignant.

Since this study employed both CNN-based models (e.g., ResNet50, VGG19) and Transformer-based models (e.g., ViT/Deit), slightly different preprocessing conventions were applied to ensure compatibility. For CNN architectures pretrained on ImageNet, images were resized to 224 × 224 × 3 pixels and normalized to the [0, 1] range by dividing each pixel by 255. For Vision Transformer models, we followed the Hugging Face preprocessing convention, where images were represented as 3 × 224 × 224 tensors and passed through the patch embedding layers of the transformer. In both cases, the classification head was excluded (include_top = False for CNNs), and uniform classification layers were added to maintain consistency across the individual models and the ensemble pipeline. This standardization facilitates efficient training, ensures architectural compatibility, and supports fair performance comparison [57].

### 3.3. Data Splitting

To ensure robust evaluation of model performance, the dataset was divided into training (80%) and testing (20%) subsets. This stratified split supports effective training of AI models while preserving representative class distributions across both subsets. The split is designed to facilitate multi-class classification tasks, distinguishing between normal, benign, and malignant cases.

### 3.4. Data Augmentation

In the field of medical imaging—particularly in breast ultrasound—labeled datasets are often limited, making data augmentation a critical strategy for enhancing the generalization ability of deep learning models. By introducing controlled variations to training images, data augmentation helps prevent overfitting and encourages the learning of more robust and invariant features. Techniques such as rotation, flipping, contrast adjustment, cropping, and zooming have consistently demonstrated effectiveness in enhancing classification performance across various studies [58,59,60].

In this study, a comprehensive runtime augmentation pipeline was implemented using TensorFlow’s Keras API (Google Brain, Mountain View, CA, USA), version 2.10.0. The augmentation techniques applied during the training phase included the following transformations:

- Resizing: Images were resized to match the input resolution required by the pre-trained feature extractor models.
- Random flipping: Horizontal and vertical flips were applied with a probability of 0.5 to simulate variability in lesion orientation.
- Random rotation: A rotation factor of 0.2 was used, allowing for image rotations of up to ±36 degrees, simulating the potential rotation of ultrasound images.
- Random contrast adjustment: A contrast factor of 0.2 was applied to simulate variations in image intensity and lighting conditions.
- Random cropping: A target height and width of 20% of the original dimensions were used to introduce local occlusions and simulate positional variability in lesions.
- Random zooming: Zoom transformations with both height and width factors set to 0.2 were used to reflect differences in imaging distance and magnification.

These augmentations were implemented using TensorFlow’s Sequential data augmentation layer, ensuring reproducibility and consistency throughout the training process. This approach significantly enhanced the model’s ability to generalize to unseen cases by introducing sufficient variability into the training data while preserving essential anatomical features. Prior research has demonstrated the effectiveness of such augmentation strategies in improving the performance of deep learning models in image classification tasks [61,62].

### 3.5. The Proposed Deep Learning Models

In this study, breast ultrasound (BUS) images are classified using three categories of models: individual convolutional neural networks (CNNs), Vision Transformer (ViT)-based models, and ensemble models.

#### 3.5.1. AI-Based Individual Models

This study leverages a diverse collection of individual pre-trained convolutional neural network (CNN) deep learning models to classify breast ultrasound (BUS) images. All models were originally trained on the ImageNet dataset for 1000-class object recognition and subsequently fine-tuned for our specific classification tasks.

The CNN-based architectures utilized in this study include VGG16 [44], VGG19 [45], MobileNetV2 [37], ResNet50 [46], Xception [48], InceptionResNetV2 [49], DenseNet201 [47], and InceptionV3 [50].

For transformer-based models, we include ViT-Hybrid [55], ViT [23], Deit [51], Dit [52], Swin [54], and Beit [53]. These models utilize frozen transformer encoders and decoders while appending the same custom classification block. This strategy ensures uniformity in training across architectures while leveraging the high-level representation capabilities of transformers.

The vision transformer (ViT) is a deep learning encoder–decoder that weighs input data for image recognition [63]. ViT extracts features from input data to improve object identification accuracy [64]. The Vision Transformer (ViT) model processes input images by linearly flattening 16 × 16 2D image patches into 1D vectors. These vectors are then input into a transformer encoder, which consists of Multi-Layer Perceptron (MLP) blocks and multi-head self-attention (MSA) mechanisms. MSA’s attention mechanism is calculated by Equation (1).

(1) $$Attention\left(Q,K,V\right)=Softmax\left(\frac{Qk^{t}}{\sqrt{d_{k}}}\right)v,$$

In this context, $d_{k}$ represents keys of dimension, K denotes the key vector, Q the query vector, and V is the value-dimensional vector. Moreover, the scaling factor $\sqrt{d_{k}}$ is included to stabilize gradients during training and prevent excessively large dot-product values, ensuring numerical stability in SoftMax computation [65]. Meanwhile, multi-head attention allows the model to process inputs from different representation subspaces concurrently. Equation (2) shows how multi-head attention employs multiple learned linear projections to linearly extend queries, keys, and values.

(2) $$MultiHead\left(Q,K,V\right)=Concat\left({head}_{1},\ldots ,{head}_{h}\right)W^{o};headi\;=Attention\left({QW}_{i}^{Q},{KW}_{i}^{K},{VW}_{i}^{V}\right),$$

where the projection matrices are $W^{o}\in R^{{hd}_{v}xd_{model}}$, $W_{i}^{Q}\in R^{d_{model}xd_{k}},W_{i}^{K}\in R^{d_{model}xd_{k}}$, and $W_{i}^{V}\in R^{d_{model}xd_{v}}$. The ‘vit-base-patch16-224-in21k’ pre-trained model, trained on 14 million images to classify 21,843 classes [63], is employed in this study. For classification purposes, we add a 1024-neuron layer, batch normalization, a 50% dropout layer, and a dense layer with 3 neurons. Importantly, all layers except the classification layers are frozen.

#### 3.5.2. AI-Based Ensemble of Individual Models

Ensemble learning has been widely applied in various studies to enhance classification performance by integrating multiple individual models through a concatenation layer [23,44,46]. This approach leverages the strengths of different models, ultimately improving the overall predictive accuracy. By combining distinct classifiers, more valuable information is extracted, leading to more precise classification results.

In this study, three ensemble-based models were constructed: (VGG19 + ResNet50), (VGG19 + ResNet50), and (DenseNet201 + ResNet50). These models were selected based on their superior performance compared to other individual classifiers, as demonstrated in the Results Section. Additionally, to explore potential improvements, we incorporated ensemble architectures previously used in breast cancer detection via mammograms, such as (DenseNet201 + VGG16 + Xception) and (DenseNet201 + VGG16 + InceptionResNetV2) [44,66] for breast cancer detection using mammograms. Building upon this, we introduced two additional ensemble models: (DenseNet201 + VGG19 + Xception) and (DenseNet201 + VGG19 + InceptionResNetV2).

To construct the ensemble models, the classification layers of the individual models are removed, allowing them to function solely as feature extractors. This approach enables the integration of multiple models to leverage their learned representations effectively. Subsequently, new classification layers with a uniform configuration are added to ensure consistency across all ensemble models. The classification architecture consists of four layers: a 1024-neuron fully connected layer, followed by batch normalization, a 50% dropout layer to prevent overfitting, and a dense output layer with three neurons for final classification. This standardized configuration ensures fair performance evaluation while enhancing the ensemble model’s ability to generalize across diverse data distributions.

#### 3.5.3. AI-Based Ensemble of Deit and ViT Model

This study introduces a novel ensemble model by merging two high-performing transformer architectures, Deit and ViT, using feature-level concatenation [23,44,46,67]. Both models are first pre-trained and then converted into fixed feature extractors by removing their original classification layers. The extracted features are concatenated and passed through a standardized classification module consisting of four layers, as detailed previously.

The rationale for this ensemble lies in the complementary strengths of the two models:

- ViT excels at capturing global contextual relationships through pure self-attention mechanisms, enabling robust high-level feature abstraction [63].
- Deit enhances model efficiency through knowledge distillation and data optimization techniques, demonstrating superior performance in limited-data scenarios [68].

By concatenating features from both Deit and ViT, the proposed ensemble leverages the rich global contextual representations of ViT alongside the enhanced generalization capability of Deit, resulting in more diverse and discriminative feature embeddings [68].

### 3.6. Fine-Tuning Models

To tailor pre-trained deep learning models for the classification of breast ultrasound (BUS) images, a systematic fine-tuning strategy was adopted. Initially, the earlier layers—responsible for capturing low-level, general features—were frozen to retain the benefit of pre-learned visual representations from large-scale datasets. Subsequently, deeper layers were selectively unfrozen to enable adaptation to domain-specific characteristics of BUS imagery.

For CNN-based models, fine-tuning began at specific layer indices near the classification block, allowing the network to adjust higher-level features for the target task. In contrast, for transformer-based and ensemble models, only the appended classification head was made trainable, with the feature extraction layers kept frozen. This differential strategy preserved the strengths of each architecture while enabling task-specific learning.

To ensure uniform evaluation and fair comparison across all model architectures, a consistent classification head was appended to each pre-trained backbone. This classification block included the following:

- A fully connected (dense) layer with 1024 neurons.
- A batch normalization layer to stabilize learning.
- A dropout layer with a rate of 0.5 to reduce overfitting.
- A final dense output layer whose configuration is task-dependent:
  ➢ 3 neurons for multiclass classification (normal, benign, malignant),
  ➢ 2 neurons for binary classification (benign vs. malignant),
  ➢ 4 or 6 neurons for BI-RADS scoring, depending on the dataset used.

This standardized design provides a consistent foundation for evaluating and comparing model performance across different model families and classification scenarios.

To streamline presentation and minimize redundancy, Table 1 summarizes the model configurations, including architecture variants, fine-tuning depth, input resolution (224×224), and classification head design.

### 3.7. Environment Setup

In this study, experiments were conducted on an ASUS laptop equipped with an AMD Ryzen 9 5900HX processor (16 cores, 3.3 GHz), 32 GB of RAM, and an NVIDIA GeForce RTX 3080 GPU with 16 GB of VRAM. The deep learning models were implemented in a Jupyter Notebook environment using Python 3.8.0, running on Windows 11. TensorFlow and Keras were utilized as the primary deep learning frameworks, offering robust functionality for model development, training, and evaluation. This hardware–software configuration provided the computational capacity necessary for efficiently processing large-scale breast ultrasound image datasets and optimizing deep learning architectures.

Model training was performed for up to 200 epochs using the AdamW optimizer, with a learning rate of 0.0001 and a weight decay factor of 4 × 10^−5^. Early stopping was employed with a patience threshold of 50 epochs to prevent overfitting and enhance training efficiency.

## 4. Results

To comprehensively evaluate the effectiveness of deep learning models for breast ultrasound image classification, this study is structured into three experimental scenarios using the BUSI dataset. In addition, the evaluation incorporates a fusion-concatenation strategy (feature-level fusion), feature visualization with t-distributed Stochastic Neighbor Embedding (t-SNE), and quantitative measures such as Silhouette Score and inter-class distance metrics.

- Scenario A investigates traditional CNN-based models. Eight popular pre-trained architectures—VGG19, VGG16, MobileNetV2, ResNet50, Xception, InceptionResNetV2, DenseNet201, and InceptionV3—are fine-tuned for a 3-class classification task (benign, malignant, normal).
- Scenario B examines six cutting-edge transformer-based models: ViT-Hybrid, ViT, Deit, DiT, Swin, and Beit. These models are unified under a consistent classification framework and evaluated on the same task.
- Scenario C focuses on ensemble learning. It introduces a novel ViT + Deit ensemble that exploits complementary transformer features for improved classification performance. This ensemble is compared against seven CNN-based ensembles, including combinations like DenseNet201 + VGG19 + Xception, VGG16 + ResNet50, and others.

To further validate robustness and generalizability, an ablation study is conducted. This includes evaluations across multiclass, binary, and BI-RADS classification tasks, using 5-fold cross-validation across three benchmark datasets: BUSI, BUS-BRA, BrEaST, and BUSI_WHU.

### 4.1. Feature Space Analysis

**Figure 3:**
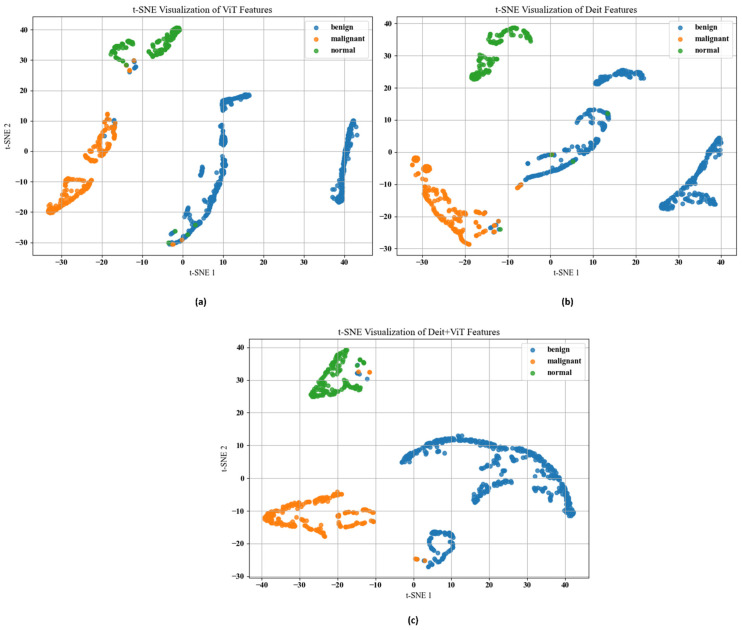
t-SNE visualization of feature spaces for (**a**) ViT, (**b**) Deit, and (**c**) the proposed Deit + ViT ensemble applied on the BUSI dataset. The ensemble (**c**) forms tighter and more distinct clusters for malignant, benign, and normal classes, with reduced overlap compared to the individual models, highlighting the effectiveness of complementary feature fusion.

To assess the discriminative quality of the learned representations, we conducted a feature visualization study using t-distributed Stochastic Neighbor Embedding (t-SNE) [23]. This analysis was applied to features extracted from the ViT, Deit, and the proposed Deit + ViT ensemble models on the BUSI dataset. As illustrated in Figure 3, the ViT model (A) demonstrates some initial class separation; however, there remains a noticeable overlap between benign and malignant categories. In contrast, the ensemble model (C) exhibits more distinct and compact clustering of classes, with minimal inter-class overlap. This suggests that the ensemble effectively captures complementary feature representations from both backbone networks, resulting in improved class separability.

For the feature fusion process, we adopted a straightforward concatenation approach rather than more complex methods such as attention-based fusion or weighted averaging. This choice was driven by the method’s simplicity, ease of interpretation, and consistent performance improvements observed during our initial experiments. To further verify the effectiveness of this fusion strategy, we quantitatively analyzed the feature-space structure using the Silhouette Score and inter-class distance metrics [69,70]. As summarized in Table 2, the Deit + ViT ensemble achieves a Silhouette Score of 0.72, indicating a significant improvement in the separability of class clusters and supporting its potential for reliable clinical application.

### 4.2. Scenario A: Breast Cancer Classification Using Individual AI Models

This experiment evaluates the performance of eight individual deep learning models on the BUSI dataset across three classes: benign, malignant, and normal. The models include VGG19, VGG16, MobileNetV2, ResNet50, Xception, InceptionResNetV2, DenseNet201, and InceptionV3. Their classification metrics are summarized in Table 3.

Among the models, ResNet50 achieved the highest performance, with an accuracy of 88.54% and an AUC of 91.65%, indicating strong discriminative capability. VGG16, VGG19, and DenseNet201 showed competitive results, each reaching an accuracy of 86.62% and AUC values of 88.14%, 88.24%, and 87.98%, respectively. In contrast, InceptionResNetV2 performed the poorest, with 70.70% accuracy and 74.72% AUC, showing difficulty in classifying between classes. Moderate accuracy performance was observed for MobileNetV2, Xception, and InceptionV3, with accuracies of 78.98%, 80.89%, and 80.89%, respectively.

**Figure 4:**
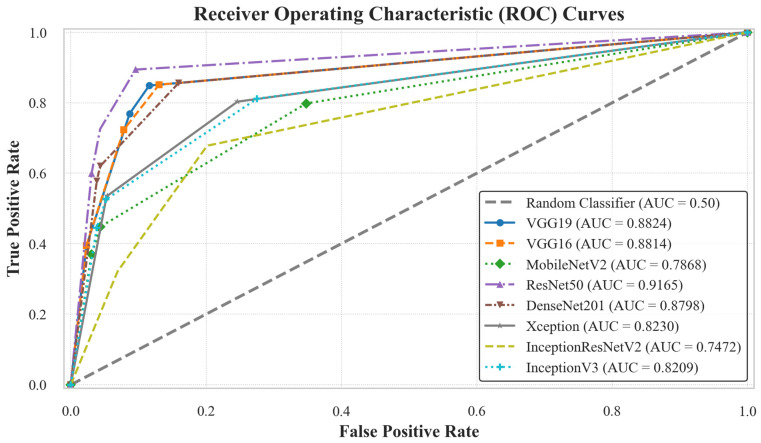
AUC curves for the individual AI models, illustrating their classification performance on the BUSI dataset. The ResNet50 model achieved the highest performance with an AUC of 91.51%.

**Figure 5:**
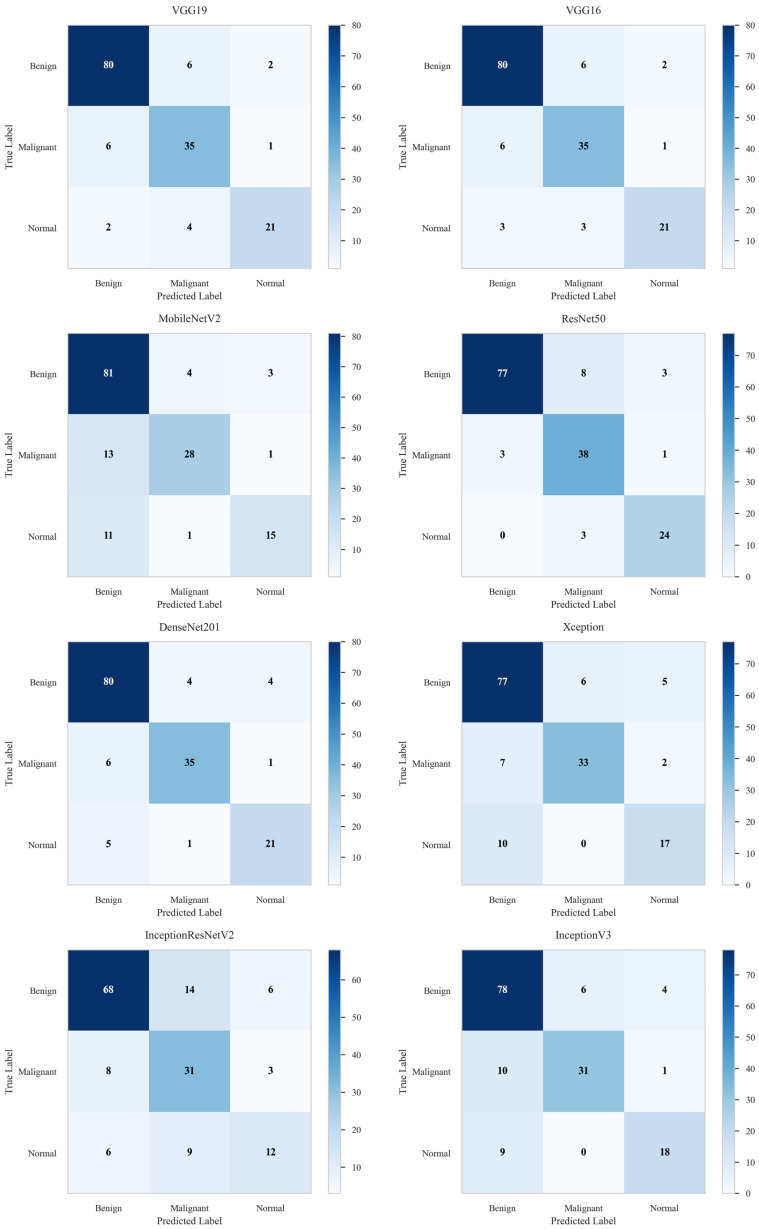
Confusion matrices of the individual AI classifiers, illustrating correctly and incorrectly classified breast ultrasound images from the BUSI dataset. The ResNet50 model demonstrated the best performance, with only 18 misclassified images out of a total of 157.

The classification performance of all individual models is illustrated in Figure 4. The AUC curves provide a comparative view of each model’s ability to discriminate between the three classes. Among them, ResNet50 achieved the highest AUC (91.65%), whereas InceptionResNetV2 recorded the lowest (74.72%). The corresponding confusion matrices further highlight model-specific misclassification patterns, as shown in Figure 5. ResNet50 demonstrated the best performance with only 18 misclassified cases out of 157, while VGG16, VGG19, and DenseNet201 each misclassified 21 samples. In contrast, MobileNetV2, Xception, and InceptionV3 misclassified 33, 30, and 30 cases, respectively, whereas InceptionResNetV2 exhibited the weakest performance with 46 misclassifications.

### 4.3. Scenario B: Breast Cancer Classification Using Vision Transformer Models

In this scenario, six Vision Transformer (ViT)-based architectures were evaluated to determine the most effective model for classifying breast ultrasound images. Among them, the standard ViT model achieved the highest performance, with an accuracy of 93.63% and an AUC of 93.98%. The Deit model followed closely, attaining 91.72% accuracy and 92.64% AUC, as detailed in Table 4.

Other models, including ViT-Hybrid, Dit, Swin, and Beit, demonstrated varied performance. Swin and Beit performed comparably, each reaching 90.45% accuracy, with AUCs of 91.89% and 90.63%, respectively. ViT-Hybrid and Dit achieved lower accuracies of 86.62% and 84.71%, with corresponding AUCs of 88.02% and 86.14%.

**Figure 6:**
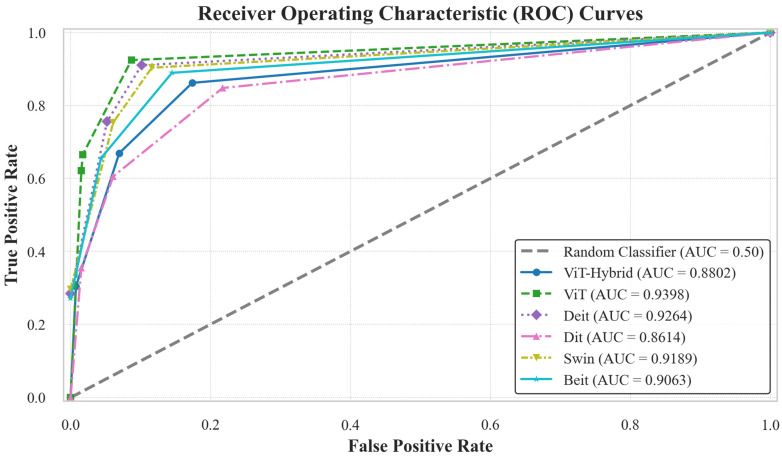
AUC curves of the Vision Transformer models. This figure illustrates the classification performance of various ViT-based models on the BUSI dataset. The ViT model achieved the best performance with the highest AUC of 93.98%.

**Figure 7:**
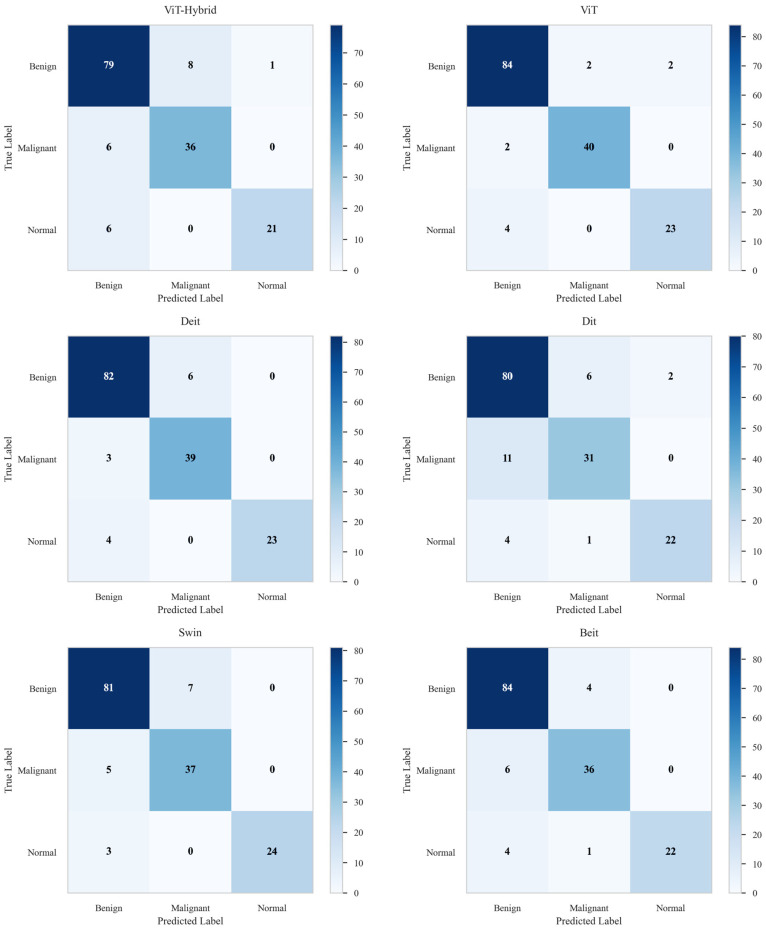
Confusion matrices of Vision Transformer models on the BUSI dataset. The ViT and Deit models demonstrated the best performance, with only 10 and 13 misclassifications, respectively, out of the total test set.

Furthermore, as shown by the AUC curves in Figure 6, the ViT classifier demonstrates the best performance with a value of 93.98%. This is further supported by the confusion matrices in Figure 7, which reflect the models’ robustness. The ViT and Deit models exhibited the fewest misclassifications at 10 and 13 cases, respectively. This highlights their superior ability to accurately distinguish between the normal, benign, and malignant classes of breast ultrasound images. In comparison, ViT-Hybrid, Dit, Swin, and Beit models misclassified a notably higher number of instances, with 21, 24, 15, and 15 misclassifications.

### 4.4. Scenario C: Breast Cancer Classification-Based AI Ensemble Classifier

In this scenario, ensemble models are evaluated using classification layers configured identically to those employed in the individual CNN and transformer-based models, ensuring fair and consistent performance comparison across all architectures.

Table 5 presents the evaluation metrics for the selected ensemble models. Among them, the Deit + ViT ensemble achieves the best overall performance, with an accuracy of 94.27% and an AUC of 94.81%. Notably, the two-model ensembles—VGG16 + ResNet50, VGG19 + ResNet50, and DenseNet201 + ResNet50—achieve average accuracies and AUC scores of 91.08%/91.49%, 89.17%/89.97%, and 89.31%/91.00%, respectively. Among the three-model ensembles, the combination of DenseNet201 + VGG19 + InceptionResNetV2 demonstrates superior performance, attaining an accuracy of 90.45% and an AUC of 91.09%, surpassing other configurations in this category.

**Figure 8:**
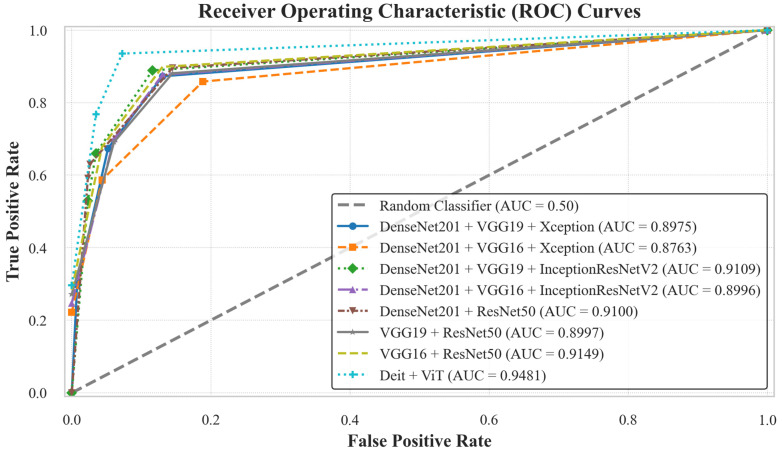
AUC curves of the proposed ensemble AI models on the BUSI dataset. This figure compares the classification performance of various ensemble models. The proposed Deit + ViT ensemble demonstrated superior performance by achieving the highest AUC of 94.81%, outperforming all other suggested ensembles.

**Figure 9:**
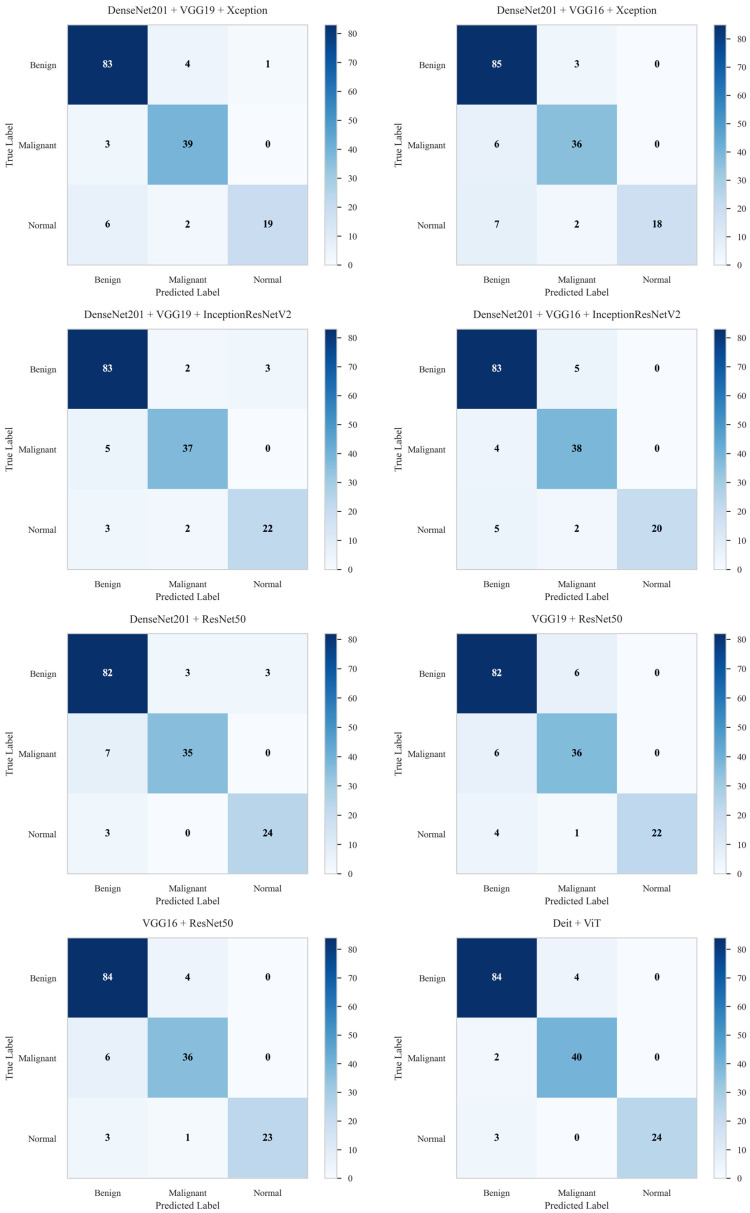
Confusion matrices of the proposed ensemble models on the BUSI dataset. The Deit + ViT ensemble demonstrated superior performance by correctly classifying the highest number of samples, resulting in the lowest number of misclassifications: only 9 out of 157 test samples. This outstanding performance highlights its effectiveness compared to other state-of-the-art models.

The AUC curves in Figure 8 provide a visual validation of the performance hierarchy, with the proposed Deit + ViT ensemble achieving the highest AUC of 94.81%. This finding is further supported by the confusion matrix in Figure 9, which shows that the Deit + ViT ensemble misclassified only 9 of the 157 test samples, thereby demonstrating a superior level of precision compared to other ensembles. In contrast, the DenseNet201 + ResNet50, VGG19 + ResNet50, and VGG16 + ResNet50 models misclassify 16, 17, and 14 samples, respectively. Among the three-model ensembles, the best performance is achieved by the DenseNet201 + VGG19 + InceptionResNetV2 combination, which misclassifies 15 images, while the DenseNet201 + VGG16 + Xception model exhibits the poorest performance, with 18 misclassified samples.

#### 4.4.1. Detailed Analysis of Misclassified Samples for the Deit + ViT Ensemble Model

To further investigate the underlying causes of misclassification, we performed a dual similarity analysis comparing each misclassified test image with its most similar training samples. Two complementary approaches were employed:

(i) Model-based similarity, computed using cosine similarity between deep feature embeddings extracted from the trained network; and
(ii) Pixel-based similarity, computed directly from normalized raw image intensities.

This analysis provides insight into how the model internally represents ultrasound images and whether misclassified cases genuinely resemble training samples from incorrect classes.

##### Model-Based Similarity Reveals Latent Feature Overlap

Across all misclassified samples, the model-based cosine similarity between a test image and its nearest training neighbors was consistently extremely high, often exceeding 0.99, regardless of whether the retrieved samples belonged to the benign, malignant, or normal classes. This indicates that the network tends to map visually distinct ultrasound images to highly similar representations in the latent space. Rather than encoding fine-grained discriminative features, the model appears to emphasize coarse-level textural patterns that are common across all ultrasound images, such as speckle noise, shadowing artifacts, and general echotexture variations.

This observation suggests that the feature embedding space exhibits insufficient class separation, with considerable overlap between representations of benign and malignant lesions. Consequently, a benign lesion may be positioned in close proximity to malignant samples within the learned feature space—even when pixel-level differences exist—leading to classification errors.

##### Pixel-Based Similarity Confirms Visual Distinctiveness

In contrast to the near-identical similarity observed in the embedding space, pixel-based cosine similarity between the same image pairs was substantially lower, typically ranging between 0.82 and 0.88. These moderate similarity values indicate that the misclassified test images are not visually identical to the training images the model considers most similar. Thus, the high embedding similarity cannot be attributed to true visual resemblance but instead reflects the model’s compression of ultrasound images into an overly smooth, low-discriminative representation.

This discrepancy confirms that the classifier is unable to fully preserve essential visual cues such as lesion boundaries, shape irregularity, posterior acoustic features, and margin characteristics—features that are critical for differentiating benign and malignant masses.

Overall, the similarity analysis demonstrates that misclassification does not arise because a test image is visually similar to samples from the incorrect class. Instead, errors stem from representation collapse within the embedding space, where heterogeneous ultrasound patterns are compressed into a narrow region irrespective of class. This challenge is inherent to ultrasound imaging due to its high speckle noise, machine-dependent variability, and subtle inter-class differences, and highlights the need for improved feature disentanglement strategies.

##### Recommendations for Future Improvements

The findings from the similarity analysis provide clear directions for enhancing model performance. Specifically, the use of contrastive learning, metric learning, or class-separability losses (e.g., triplet loss, center loss) may help enforce a more discriminative embedding structure. Additionally, incorporating multi-scale features, radiomics-driven shape descriptors, or edge-aware modules may strengthen the model’s ability to capture lesion-specific characteristics that are currently lost during feature abstraction.

**Figure 10:**
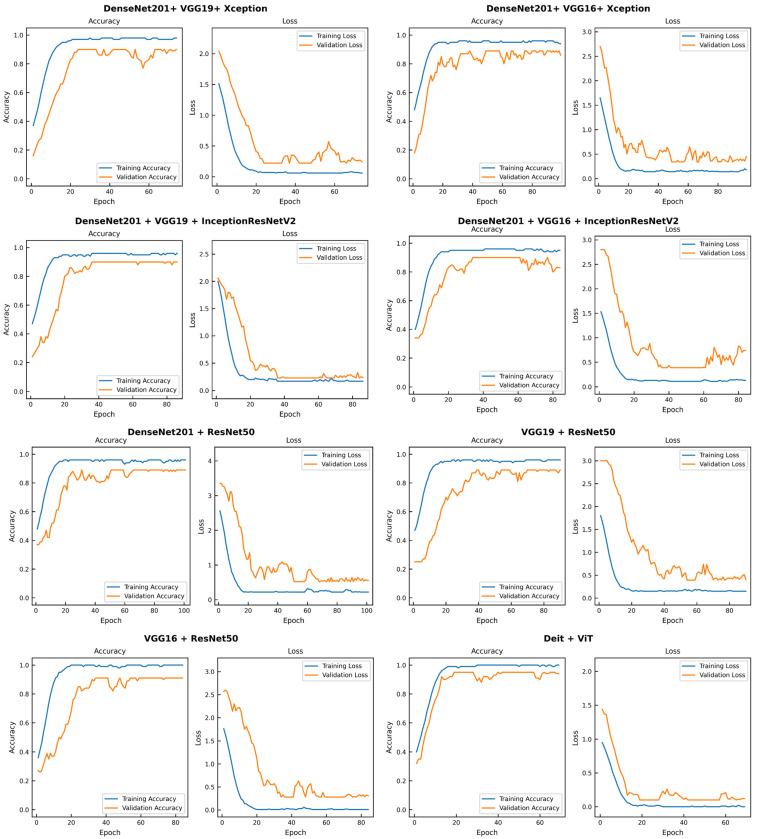
Training loss comparison of the proposed Deit + ViT ensemble model against other ensemble configurations using the BUSI dataset. The Deit + ViT ensemble exhibits a smoother and more stable convergence curve, indicating superior optimization behavior compared to the other models.

Figure 10 presents the training loss curves for all evaluated models. Notably, the proposed ensemble model exhibits a smoother and more stable convergence pattern compared to the other ensmeble architectures, indicating more consistent learning behavior. The variation in the number of training epochs across models is due to the use of an early stopping criterion (patience = 50), which terminates training once no further improvement is observed. This approach helps prevent overfitting and ensures that each model is trained only for as long as necessary.

### 4.5. Ablation Study

This section presents an ablation study conducted to assess the performance and individual contributions of the components within the proposed ensemble model, which integrates Deit and ViT transformer architectures. The objective is to determine the value added by the ensemble strategy compared to its standalone components and other widely adopted deep learning models.

To ensure a rigorous and unbiased evaluation, a 5-fold cross-validation approach was applied using the BUSI dataset. The dataset was randomly divided into five equal parts, where each fold involved training on four subsets and testing on the remaining one. This procedure was iterated across all five folds to minimize overfitting and offer a comprehensive view of the model’s generalization performance.

The proposed Deit + ViT ensemble was evaluated against four benchmark models: ResNet50, ViT, Deit, and a hybrid VGG16 + ResNet50 architecture, since they achieve the best performance across three evaluation scenarios (A, B, C). As summarized in Supplementary Table S2, the ensemble consistently outperformed all baseline models, achieving the highest average accuracy of 93.12% and AUC of 93.54%, highlighting its superior classification capability on the BUSI dataset.

To evaluate the generalizability of the proposed Deit + ViT ensemble model in more diverse and clinically realistic settings, we extended our experimental analysis to two additional benchmark datasets: BUS-BRA (Rio de Janeiro, Brazil) [71] and BrEaST (medical centers, Poland) [72]. The BUS-BRA dataset consists of 1875 de-identified ultrasound images collected from 1064 patients, including 1286 benign and 607 malignant cases, with 722 benign and 342 malignant cases confirmed via biopsy. In comparison, the BrEaST dataset comprises 256 ultrasound scans categorized into 154 benign, 98 malignant, and 4 normal cases. Both datasets include rich metadata such as BI-RADS classifications, histopathological outcomes, and expert-generated segmentations, providing a strong foundation for comprehensive model evaluation.

We assessed the ensemble model’s performance across two tasks: binary classification (distinguishing between benign and malignant lesions) and multi-class classification based on BI-RADS categories. To ensure a fair and consistent evaluation process, each dataset was split into 80% training and 20% testing subsets, aligning with the strategy used for the BUSI dataset.

In the binary classification task, the ensemble model demonstrated strong cross-dataset generalization capability. It achieved 96.92% accuracy and an AUC of 97.10% on the BUSI dataset. On the BrEaST dataset, the model maintained competitive results, reaching 87.76% accuracy and 88.07% AUC. Similarly, on the BUS-BRA dataset, the model recorded 86.77% accuracy and 85.90% AUC. These findings, summarized in Table 6, highlight the robustness and adaptability of the proposed approach across varied imaging environments and patient demographics. Moreover, another dataset called BUS_WHU (Renmin Hospital of Wuhan University, China) was used to test the proposed model, which consisted of 560 benign and 367 malignant images [73]; however, we used 367 benign images and randomly selected 367 malignant images to create a balanced dataset. Consistently, the ensemble model exhibits superior performance, reaching an accuracy of 86.99% and an F1-score of 86.98%, demonstrating its generalization stability across different datasets issued from various modalities.

In the more complex multi-class classification task based on BI-RADS categories, the performance of the proposed Deit + ViT ensemble model was comparatively moderate. On the BUS-BRA dataset, which spans BI-RADS categories 2 through 5, the model achieved an accuracy of 76.68% and an AUC of 84.59%. For the BrEaST dataset, which features a more detailed classification scheme including BI-RADS categories 2, 3, 4a, 4b, 4c, and 5, the model attained an accuracy of 68.75% and an AUC of 81.10%, as summarized in Table 7.

While the ensemble model demonstrates excellent performance in binary classification (benign vs. malignant) across all datasets, its accuracy in multi-class BI-RADS classification is comparatively lower. This outcome is expected, as BI-RADS assignments are inherently subjective and depend on the radiologist’s expertise, whereas binary classification directly corresponds to histology—the clinical gold standard. Therefore, the binary classification task provides a more objective and clinically meaningful evaluation of model performance.

**Figure 11:**
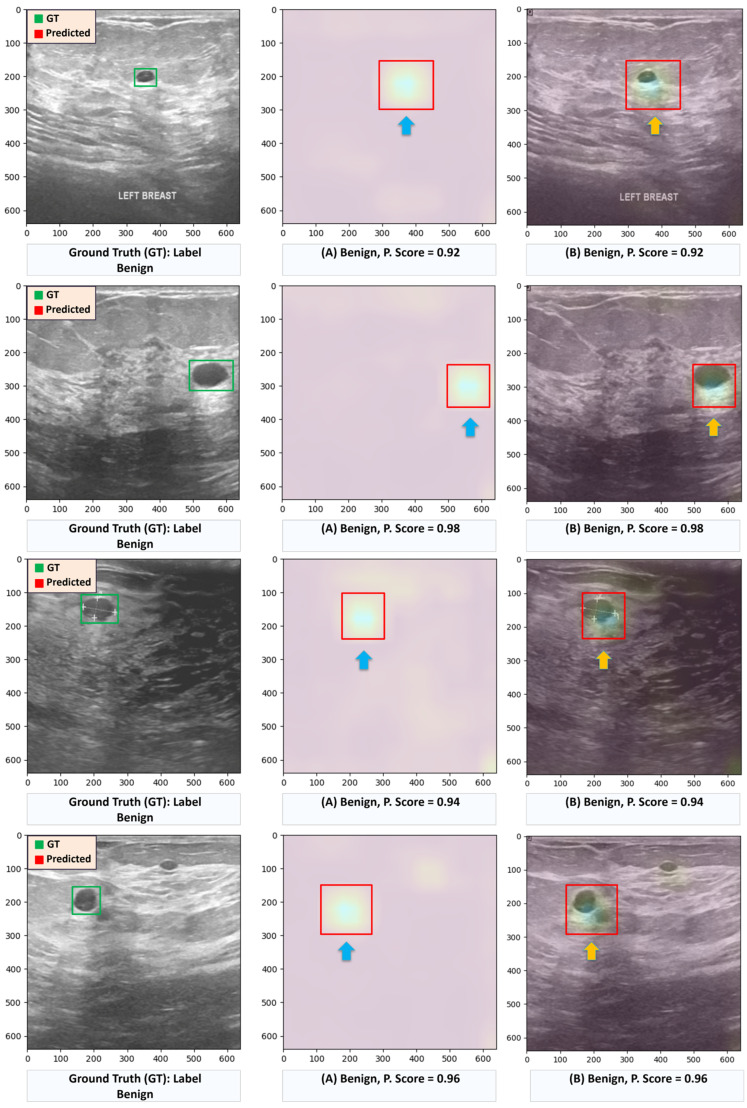
Examples of benign cases from the BUSI dataset illustrating regions highlighted by the proposed ensemble Deit + ViT model. (A) Regions of interest with the background removed, marked with rectangles to indicate suspected lesion areas. (B) Original image overlaid with Grad-CAM heatmaps; arrows and rectangles correspond to the suspected lesion locations for direct visual comparison.

To enhance the interpretability and validate the decision-making process of the proposed ensemble-based Deit and ViT model, the Grad-CAM technique [23] was employed. This analysis, conducted using the BUSI dataset, aims to reveal the critical regions the model focuses on when classifying breast ultrasound images. As shown in Figure 11, heatmaps derived from the model’s final convolutional layer highlight the regions of interest (ROIs) associated with potential lesions.

Specifically, two benign cases are analyzed to illustrate the model’s behavior, with predicted probability scores (P Scores) indicating the confidence in benign classification. For each case, two visual representations are presented: the first pairs the Grad-CAM heatmap with the original image, allowing the ROI to be isolated and enclosed within a bounding box; the second directly overlays the heatmap onto the ultrasound image, offering a more intuitive and clinically interpretable view of the model’s attention.

These visual explanations confirm a strong correspondence between the model’s focus areas and the ground truth (GT) annotations, reinforcing the model’s reliability and interpretability—key factors for clinical applicability and trust in AI-assisted diagnostics.

**Figure 12:**
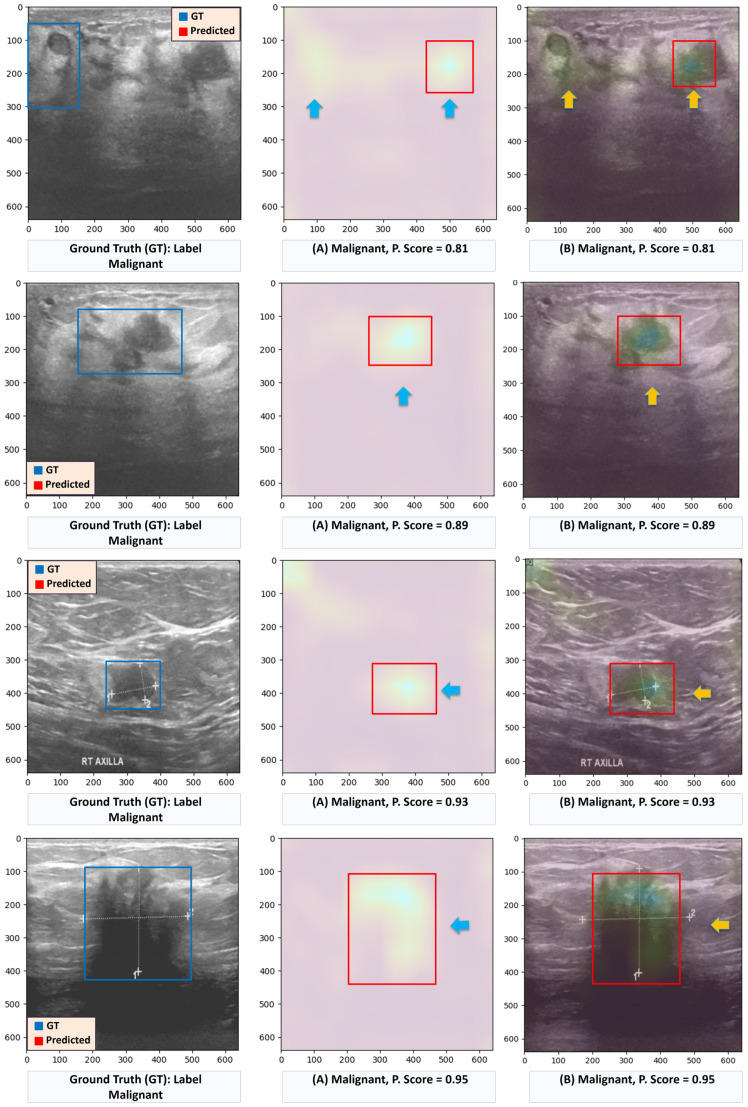
Examples of malignant cases from the BUSI dataset showing regions highlighted by the proposed ensemble Deit + ViT model. Regions of interest with the background removed and rectangles marking the suspected lesion areas. Original image overlaid with Grad-CAM heatmaps; arrows and rectangles indicate the detected regions corresponding to model attention.

Figure 12 further demonstrates the model’s capability to localize regions of interest (ROIs) in malignant cases. In contrast to benign cases, the accuracy of the highlighted regions is somewhat reduced, likely due to the greater complexity and heterogeneity characteristic of malignant tumors. Although the model may identify multiple activated regions, the largest highlighted area is used to define the predicted bounding boxes, ensuring a focused and consistent representation of the model’s primary attention.

These results underscore the superior performance of the ensemble-based Deit and ViT model, showcasing its ability to effectively capture complex global dependencies in the input data. By integrating the complementary strengths of both transformer architectures, the ensemble achieves high classification accuracy and consistent robustness across various evaluation metrics. This synergy highlights the advantages of model fusion, leading to more refined and reliable predictions.

## 5. Discussion

### 5.1. Performance Evaluation of the Proposed AI Models

This study evaluated three experimental scenarios to assess CNN-, transformer-, and ensemble-based models for breast ultrasound classification using the BUSI dataset.

In Scenario A, eight pre-trained CNNs were compared. ResNet50 achieved the best performance (88.54% accuracy, 91.50% AUC), while InceptionResNetV2 showed the weakest results. VGG16, VGG19, and DenseNet201 also performed competitively, and these top CNNs were selected as baseline ensembles for comparison with transformer-based models.

In Scenario B, transformer architectures substantially outperformed CNNs. Deit (93.63% accuracy, 94.01% AUC) and ViT (91.72% accuracy, 92.31% AUC) demonstrated strong feature representation and motivated the development of a combined Deit–ViT ensemble.

In Scenario C, ensemble models were compared. Although the CNN-based VGG16 + ResNet50 ensemble performed reasonably well (91.08% accuracy), the proposed Deit + ViT ensemble achieved the highest performance (94.27% accuracy, 94.81% AUC) with the fewest misclassifications. These findings confirm the advantage of combining transformer architectures for complex ultrasound classification.

Remaining misclassifications were largely associated with image quality issues (noise, low contrast), class imbalance, and challenging borderline lesions difficult to separate visually. These challenges should be addressed in future dataset expansion and model refinement.

In summary, the proposed Deit + ViT ensemble, constructed via a concatenation-based fusion layer, outperforms individual models and other ensemble strategies across multiple evaluation metrics. Although this approach incurs a modest increase in training time, the resulting performance gains justify the computational overhead—especially as hardware continues to advance.

### 5.2. Clinical Applicability and Deployment Considerations

Although technically robust, the model’s deployment in clinical practice requires attention to workflow integration, explainability, and regulatory pathways.

The proposed CAD system can serve as a second-reader tool, assisting radiologists in high-volume or resource-limited settings. Integration through PACS-compatible APIs would allow seamless access to predictions without disrupting routine radiology workflows. Explainability techniques such as Grad-CAM provide essential transparency and help clinicians validate model outputs.

However, current limitations related to BI-RADS interpretation remain. Incorporating BI-RADS-labeled datasets and structured ultrasound reports would align the model more closely with clinical diagnostic standards.

Clinical deployment also requires further multi-center validation, adherence to regulatory requirements, and a human-in-the-loop framework to ensure safety and reliability.

### 5.3. The Complexity Time of the Proposed CAD Framework

The proposed ensemble CAD framework was evaluated for its computational efficiency (Table 8), considering key metrics like trainable parameters, training time per epoch, inference time per image, and image throughput (FPS) on the BUSI dataset. Although the model requires greater computational resources and exhibits higher time consumption during both training and inference, achieving a relatively lower image throughput of 31.25 FPS compared to some state-of-the-art models, this trade-off is justified by its superior performance across all evaluation metrics, highlighting its strong classification accuracy, robustness, and suitability for clinical tasks, where diagnostic quality is prioritized over raw processing speed.

### 5.4. Comparison with Related Work on Breast Cancer Classification

This section presents a comparative evaluation of the proposed ensemble-based Deit and ViT transformer model against recent state-of-the-art research using the BUSI dataset in breast cancer classification, as summarized in Table 9. This ensemble achieves superior performance to most reported methods, in both multi-class and binary classifications. Although direct comparisons are limited by differences in data preprocessing and evaluation protocols, the consistently strong performance demonstrates the model’s potential for real-world diagnostic support.

### 5.5. Limitation and Future Work

Despite the promising performance of the ensemble framework for classifying breast ultrasound images, several limitations and opportunities for future work exist. First, the study used a limited set of deep learning architectures, and while the Deit and ViT ensemble performed best, incorporating models like ResNet-based Vision Transformers [74,75,76,77] could further boost accuracy, especially since models like RegNet [78] (85.99%) and Levit [79] (56.05%) were excluded due to suboptimal results on the BUSI dataset. Second, the current dataset suffers from imbalance and variability, making the collection of larger, balanced, and diverse datasets a key future goal to improve generalizability. Third, future research will move beyond simple feature concatenation to explore adaptive fusion strategies, such as attention-based mechanisms, to dynamically reweight features for complex diagnostic scenarios. Fourth, to enhance clinical applicability and address the framework’s reduced performance under the BI-RADS classification scheme, future work will integrate structured radiology reports and metadata (patient and scanner info) from clinical datasets to provide context-specific features [80]. Finally, the integration of Large Language Models (LLMs) [81] may facilitate medical data interpretation and enhance BI-RADS predictions, making dataset expansion essential for robust multiclass classification and improved real-world performance.

## 6. Conclusions

Early detection of breast cancer is crucial in reducing mortality rates globally. This study introduces a novel Computer-Aided Diagnosis (CAD) system that utilizes an ensemble of transformer-based models—Vision Transformer (ViT) and Data-efficient Image Transformer (Deit)—integrated through transfer learning to enhance feature extraction and classification. The architecture combines discriminative features from both models using a concatenation layer, followed by convolutional neural network (CNN) layers to classify breast ultrasound images into normal, benign, malignant, or BI-RADS categories.

To ensure consistency and minimize bias, classification layers were kept uniform across all experiments. Data augmentation techniques—random flipping, rotation, and zooming—were applied during training to improve generalization. Alongside the proposed ensemble, a range of state-of-the-art models, including VGG16, VGG19, MobileNetV2, ResNet50, Xception, InceptionV3, InceptionResNetV2, DenseNet201, ViT-Hybrid, Swin, and Beit were benchmarked using the BUSI dataset.

The ensemble model demonstrated excellent performance, achieving 94.27% accuracy and 94.81% AUC in multiclass classification, and 96.92% accuracy with 97.10% AUC in binary classification on the BUSI dataset. Through 5-fold cross-validation, the Deit + ViT ensemble consistently outperformed individual models and hybrid CNN baselines, with the highest average accuracy (93.12%) and AUC (93.54%).

External validations on the BUS-BRA, BrEaST, and BUSI_WHU datasets further confirmed the model’s robustness, with AUCs of 85.90%, 88.07%, and 86.99% in binary classification, respectively. While results for BI-RADS multiclass classification were encouraging, further work is needed to improve performance on fine-grained clinical labels.

These findings underscore the potential of transformer-based ensemble learning in ultrasound-based breast cancer diagnosis. The proposed CAD system offers a reliable, interpretable, and clinically relevant tool to assist radiologists. Future efforts will focus on regulatory validation, seamless integration into clinical workflows, and enhancing explainability. Expanding to additional imaging modalities and diverse, multi-center datasets will further strengthen its real-world applicability across various healthcare settings.

## Figures and Tables

**Table 1:** Overview of deep learning models and their corresponding fine-tuning strategies.

Model	Pre-Trained Variant	Trainable Layers (From Index)	Custom Classification Layers
VGG16	ImageNet	From layer 17	1024 FC → BatchNorm → Dropout (0.5) → Dense (3)
VGG19	ImageNet	From layer 17	Same as above
MobileNetV2	ImageNet	From layer 131	Same as above
ResNet50	ImageNet	From layer 123	Same as above
Xception	ImageNet	From layer 96	Same as above
InceptionResNetV2	ImageNet	From layer 720	Same as above
InceptionV3	ImageNet	From layer 252	Same as above
DenseNet201	ImageNet	From layer 481	Same as above
ViT-Hybrid	vit-hybrid-base-bit-384	Frozen	Same as above
DIT	dit-base-finetuned-rvlcdip	Frozen	Same as above
Swin	swin-tiny-patch4-window7-224	Frozen	Same as above
Beit	beit-base-patch16-224-pt22k-ft22k	Frozen	Same as above
Deit	deit-base-patch16-224	Frozen	Same as above
ViT	vit-base-patch16-224-in21k	Frozen	Same as above
Ensemble 1	DenseNet201 + VGG19 + Xception	Frozen and used as Feature extractor only	Shared: 1024 FC → BatchNorm → Dropout (0.5) → Dense (3)
Ensemble 2	DenseNet201+ VGG16+ Xception	Frozen and used as Feature extractor only	Same as above
Ensemble 3	DenseNet201 + VGG19 + InceptionResNetV2	Frozen and used as Feature extractor only	Same as above
Ensemble 4	DenseNet201 + VGG16 + InceptionResNetV2	Frozen and used as Feature extractor only	Same as above
Ensemble 5	DenseNet201 + ResNet50	Frozen and used as Feature extractor only	Same as above
Ensemble 6	VGG19 + ResNet50	Frozen and used as Feature extractor only	Same as above
Ensemble 7	VGG16 + ResNet50	Frozen and used as Feature extractor only	Same as above
The proposed Ensemble	Deit + ViT	Frozen and used as Feature extractor only	1024 FC → BatchNorm → Dropout (0.5) → Dense (2/3/4/6)

**Table 2:** Performance metrics and feature-space separability of ViT, Deit, and the proposed Deit + ViT ensemble applied on the BUSI dataset.

Model	Silhouette Score	Inter-Class Distance
ViT	0.50	1.5
Deit	0.48	1.4
Deit + ViT	0.72	2.8

Note: Silhouette Score: −1 (worst) to +1 (best); Inter-Class Distance: Higher = better separation.

**Table 3:** Experimental evaluation (%) of the selected individual AI classifiers using the BUSI dataset.

AI Model	Class	FP	Acc.	AUC	Evaluation Matrices (%)		
					PRE.	SE.	F1.
VGG19	Benign	8	86.62	88.24	91.00	91.00	91.00
	Malignant	7			78.00	83.00	80.00
	Normal	6			88.00	78.00	82.00
VGG16	Benign	8	86.62	88.14	90.00	91.00	90.00
	Malignant	7			80.00	83.00	81.00
	Normal	6			88.00	78.00	82.00
MobileNetV2	Benign	7	78.98	78.68	77.00	92.00	84.00
	Malignant	14			85.00	67.00	75.00
	Normal	12			79.00	56.00	65.00
ResNet50	Benign	11	88.54	91.65	96.00	88.00	92.00
	Malignant	4			78.00	90.00	84.00
	Normal	3			86.00	89.00	87.00
DenseNet201	Benign	8	86.62	87.98	88.00	91.00	89.00
	Malignant	7			88.00	83.00	85.00
	Normal	6			81.00	78.00	79.00
Xception	Benign	11	80.89	82.30	82.00	88.00	85.00
	Malignant	9			85.00	79.00	81.00
	Normal	10			71.00	63.00	67.00
InceptionResNetV2	Benign	20	70.70	74.72	83.00	77.00	80.00
	Malignant	11			57.00	74.00	65.00
	Normal	15			57.00	44.00	50.00
InceptionV3	Benign	10	80.89	82.09	80.00	89.00	84.00
	Malignant	11			84.00	74.00	78.00
	Normal	9			78.00	67.00	62.00

**Table 4:** Experimental evaluation (%) of the selected Vision Transformer models using the BUSI dataset.

AI Model	Class	FP	Acc.	AUC	Evaluation Matrices (%)		
					PRE.	SE.	F1.
ViT-Hybrid	Benign	9	86.62	88.02	87.00	90.00	88.00
	Malignant	6			82.00	86.00	84.00
	Normal	6			95.00	78.00	86.00
ViT	Benign	4	93.63	93.98	93.00	95.00	94.00
	Malignant	2			95.00	95.00	95.00
	Normal	4			92.00	85.00	88.00
Deit	Benign	6	91.72	92.64	92.00	93.00	93.00
	Malignant	3			87.00	93.00	90.00
	Normal	4			100.0	85.00	92.00
Dit	Benign	8	84.71	86.14	84.00	91.00	87.00
	Malignant	11			82.00	74.00	78.00
	Normal	5			92.00	81.00	86.00
Swin	Benign	7	90.45	91.89	91.00	92.00	92.00
	Malignant	5			84.00	88.00	86.00
	Normal	3			100.0	89.00	94.00
Beit	Benign	4	90.45	90.63	89.00	95.00	92.00
	Malignant	6			88.00	86.00	87.00
	Normal	5			100.0	81.00	90.00

**Table 5:** Experimental evaluation (%) of the selected ensemble models using concatenation-based fusion on the BUSI dataset.

AI Model	Class	FP	Acc.	AUC	Evaluation Matrices (%)		
					PRE.	SE.	F1.
DenseNet201 + VGG19 + Xception	Benign	5	89.81	89.75	90.00	94.00	92.00
	Malignant	3			87.00	93.00	90.00
	Normal	8			95.00	70.00	81.00
DenseNet201+ VGG16+ Xception	Benign	3	88.54	87.63	87.00	97.00	91.00
	Malignant	6			88.00	68.00	87.00
	Normal	9			100.0	67.00	81.00
DenseNet201 + VGG19 + InceptionResNetV2	Benign	5	90.45	91.09	91.00	94.00	93.00
	Malignant	5			88.00	88.00	88.00
	Normal	5			92.00	81.00	86.00
DenseNet201 + VGG16 + InceptionResNetV2	Benign	5	89.81	89.96	90.00	94.00	92.00
	Malignant	4			84.00	90.00	87.00
	Normal	7			100.0	74.00	85.00
DenseNet201 + ResNet50	Benign	6	89.31	91.00	89.00	93.00	91.00
	Malignant	7			92.00	83.00	88.00
	Normal	3			89.00	89.00	89.00
VGG19 + ResNet50	Benign	6	89.17	89.97	89.00	93.00	91.00
	Malignant	6			84.00	86.00	85.00
	Normal	5			100.0	81.00	90.00
VGG16 + ResNet50	Benign	4	91.08	91.49	90.00	95.00	93.00
	Malignant	6			88.00	86.00	87.00
	Normal	4			100.0	85.00	92.00
Deit + ViT	Benign	4	94.27	94.81	94.00	95.00	95.00
	Malignant	2			91.00	95.00	93.00
	Normal	3			100.0	89.00	94.00

**Table 6:** Experimental evaluation (%) of the proposed ensemble-based Deit + ViT model for binary classification (benign vs. malignant) across the BUSI, BUS-BRA, BrEaST, and BUSI_WHU datasets.

AI Model	Dataset	Class	FP	Acc.	AUC	Evaluation Matrices (%)		
						PRE.	SE.	F1.
Deit + ViT	BUSI	Benign	3	96.92	97.10	99.00	97.00	98.00
		Malignant	1			93.00	98.00	95.00
	BUS-BRA	Benign	30	86.77	85.70	92.00	88.00	90.00
		Malignant	25			77.00	83.00	80.00
	BrEaST	Benign	4	87.76	88.07	93.00	87.00	90.00
		Malignant	2			81.00	89.00	85.00
	BUSI_WHU	Benign	8	86.99	86.99	86.00	89.00	87.00
		Malignant	11			90.00	85.00	87.00

**Table 7:** Experimental evaluation (%) of the proposed ensemble-based Deit + ViT model for BI-RADS classification using the BUS-BRA and BrEaST datasets.

AI Model	Dataset	Class	FP	Acc.	AUC	Evaluation Matrices (%)		
						PRE.	SE.	F1.
Deit + ViT	BrEaST	2	1	68.75	81.32	71.00	83.00	77.00
		3	2			71.00	71.00	71.00
		4a	3			83.00	62.00	71.00
		4b	3			60.00	67.00	63.00
		4c	3			55.00	67.00	60.00
		5	3			86.00	67.00	75.00
	BUS-BRA	2	26	76.68	84.76	80.00	77.00	78.00
		3	20			70.00	76.00	73.00
		4	28			91.00	80.00	85.00
		5	11			45.00	65.00	53.00

**Table 8:** Computational performance metrics of top-performing AI models on the BUSI dataset.

Model	No. of Parameters (Million)	Training Time/ Epoch (Msec)	Testing Time/ Image (s)	Frame Per Second (FPS)
ResNet50	50.39	151	0.0110	90.90
ViT	87.18	218	0.0180	55.55
Deit	87.18	218	0.0180	55.55
VGG16 + ResNet50	70.17	380	0.025	40.00
The proposed CAD (ensemble of Deit + ViT)	174.36	490	0.032	31.25

**Table 9:** Comparative analysis of the proposed ensemble-based Deit and Vit model against state-of-the-art deep learning models for ultrasound breast cancer classification.

Reference	Dataset	Labels	Methodology	Accuracy (%)
Becker A.S. et al. (2018) [34]	Private dataset/BUSI	Benign/Malignant	Generic DL	96 (AUC)
Xiao T. et al. (2018) [33]	Private dataset/BUSI	Benign/Malignant	CNN	74.44 (Acc.) 78 (AUC)
Wang Y. et al. (2018) [41]	Private dataset/BUSI	Benign/Malignant	DCNN	95 (Se.)
Liao W.X. et al. (2020) [30]	Data were collected in the Peking University Third Hospital. /BUSI	Benign/Malignant	VGG19	90.38 (Acc.) 97 (AUC)
Zhang H. et al. (2020) [29]	Data were collected from different hospitals./BUSI	Benign/Malignant	InceptionV3	82.8 (Acc.) 90.5 (AUC)
Wan K.W. et al. (2021) [35]	Mendeley dataset and dataset from Baheya hospital./BUSI	Benign/Malignant/Normal	CNN	91 (Acc.)
Gu Y. et al. (2022) [27]	Data were collected from 32 hospitals./BUSI	Benign/Malignant	VGG-DCNN	86.40 (Acc.) 91.3 (AUC)
Lee S.E. et al. (2022) [40]	Private dataset/BUSI	Benign/Malignant	AI-CAD	85.4 (Acc.) 85.5 (AUC)
Alotaibi, Mohammed et al. (2023) [24]	BUSI	Benign/Malignant	VGG19	87.8 (Acc.), 83.8 (F1-score), 94.63 (AUC)
Zhang S. et al. (2023) [42]	Private dataset/BUSI	Normal/abnormal	U-NET + DenseNet	96 (Acc.) 99 (AUC)
Ejiyi, Chukwuebuka Joseph et al.(2024) [36]	BUSI	Benign/Malignant/Normal	SegmentNet	93.88 (Acc.)
Islam Rakibul et al. (2024) [32]	BUSI	Benign/Malignant/Normal	EDCNN	87.82 (Acc.)
Sahu, Adyasha et al. (2024) [37]	BUSI	Benign/Malignant	Ensemble of (AlexNet + ResNet + MobileNetV2)	94.62 (Acc.) in identifying malignancies.
Altameemi et al. (2025) [26]	BUSI	Benign/Malignant/Normal	DenseNet121 with custom CNN	89.87 (Acc.) 90.00 (F1-score) 89.87(Se.)
Carlos A. et al.(2025) [39]	BUSI	Benign/Malignant/Normal	UNet++	80.20 (Acc.)
The proposed model	BUSI	Benign/Malignant/Normal	Ensemble-based Deit + ViT	94.27 (Acc.), 93.19 (F1-s., Pre., Se.), 94.81 (AUC)
		Benign/Malignant		96.92 (Acc.), 93.18 (Pre.), 97.62 (Se.), 95.35 (F1-s.), 97.10 (AUC)

## Data Availability

All datasets used in this study are publicly available at: BUSI: https://scholar.cu.edu.eg/?q=afahmy/pages/dataset, 26 November 2025. BUS-BRA: https://aapm.onlinelibrary.wiley.com/doi/abs/10.1002/mp.16812, 26 November 2025. BrEaST: https://www.cancerimagingarchive.net/collection/breast-lesions-usg/, 26 November 2025. BUSI_WHU: https://data.mendeley.com/datasets/k6cpmwybk3/3, 26 November 2025.

## Supplementary Materials

The following supporting information can be downloaded at: https://www.mdpi.com/article/10.3390/diagnostics16040622/s1, S1.1 Data Splitting; S2.1. Evaluation Metrics; S3.1. 5-Fold Cross-Validation. Table S1. Data split of the BUSI dataset into training (80%) and testing (20%) subsets. Table S2. Experimental evaluation of best-performing AI models on the BUSI dataset using 5-fold cross-validation.
