# Supplementary material for: An Interpretable Ensemble Transformer Framework for Breast Cancer Detection in Ultrasound Images
Source: Diagnostics (Basel). 2026 Feb 20;16(4):622. doi: 10.3390/diagnostics16040622 (PMC12939200; doi:10.3390/diagnostics16040622)
Supplement: Supplementary file 1 [file diagnostics-16-00622-s001.zip › diagnostics-3917296-supplementary.pdf]

Supplementary Material S1

S1.1. Data Splitting

The distribution of samples across each class for training and testing is summarized in Table S1.

Table S1. Data split of the BUSI dataset into training (80%) and testing (20%) subsets.

Data Splitting / Class	Benign	Malignant	Normal	Total
Training	349	168	106	623
Testing	88	42	27	157

Supplementary Material S2

S2.1. Evaluation Metrics

To assess the performance of breast cancer classification models, several standard evaluation metrics are utilized, including precision, recall (sensitivity), accuracy, F1-score, and the Receiver Operating Characteristic (ROC) curve along with its Area Under the Curve (AUC) value. These metrics collectively provide a comprehensive understanding of a model's classification capabilities, particularly in distinguishing between different classification scenarios.

The metrics are mathematically defined as follows:

Precision (Pre.):

$$\text{Precision (Pre.)} = \frac{\text{TP}}{\text{TP+FP}}, \quad 1)$$

Measures the proportion of correctly predicted positive cases among all predicted positives. Recall / Sensitivity (Se.):

$$\text{Recall/Sensitivty (Se.)} = \frac{\text{TP}}{\text{TP+FN}}, \quad (2)$$

Indicates the model's ability to correctly identify actual positive cases (e.g., correctly detecting breast cancer). Accuracy (Acc.):

$$\text{Accuracy (Acc.)} = \frac{\text{TP} + \text{TN}}{\text{TP} + \text{TN} + \text{FP} + \text{FN}}, \quad (3)$$

Reflects the overall proportion of correct predictions across all classes. F1-score (F1-s.):

$$\text{F1} - \text{score (F1} - \text{s.)} = \frac{2 * \text{Precision} * \text{Sensitivity}}{\text{Precision} + \text{Sensitivity}}, \quad (4)$$

Provides a harmonic mean of precision and sensitivity, especially useful for imbalanced datasets.

Here, the components are defined as:

- TP (True Positive): Correctly identified cases of breast cancer.
- TN (True Negative): Correctly identified non-cancer cases.
- FP (False Positive): Non-cancer cases incorrectly identified as cancer.
- FN (False Negative): Cancer cases incorrectly identified as non-cancer.

In addition to these metrics, the ROC curve and corresponding AUC are employed to evaluate the classifier's ability to discriminate between classes. A higher AUC indicates stronger discriminatory performance, especially important in multi-class tasks such as distinguishing normal, benign, and malignant cases.

Supplementary Material S3

S3.1. 5-Fold Cross-Validation

Table S2. Experimental evaluation of best-performing AI models on the BUSI dataset using 5-fold cross-validation.

AI Model	Fold No	FP	Acc.	AUC	Evaluation Matrices (%)		
					PRE.	SE.	F1.
ResNet50	1	(12,5,5)	85.99	89.04	86.51	85.99	86.12
	2	(10,2,3)	90.45	92.67	90.95	90.45	90.55
	3	(14,6,4)	84.71	88.37	85.88	84.71	85.01
	4	(9,4,3)	89.81	91.50	89.94	89.81	89.84
	5	(13,3,2)	88.54	90.97	89.08	88.54	88.58
	AVG.		87.90	90.51	88.472	87.90	88.02
ViT	1	(5,3,3)	92.99	93.57	92.99	92.99	92.98
	2	(4,2,4)	93.63	94.01	93.61	93.63	93.59
	3	(7,5,5)	89.17	91.12	89.22	89.17	89.19
	4	(6,3,3)	92.36	93.04	92.61	92.36	92.40
	5	(8,5,6)	87.90	89.62	87.90	87.90	87.86
	AVG.		91.21	92.272	91.266	91.21	91.204
Deit	1	(5,3,5)	91.72	92.65	91.91	91.72	91.69
	2	(5,3,4)	92.36	93.19	92.33	92.36	92.33
	3	(6,4,6)	89.81	91.16	89.79	89.81	89.74
	4	(5,4,6)	90.45	90.86	90.87	90.45	90.38
	5	(7,6,6)	87.90	89.28	87.89	87.90	87.84
	AVG.		90.448	91.428	90.558	90.448	90.396
VGG16 + ResNet50	1	(5,4,5)	91.08	92.32	91.05	91.08	91.04
	2	(6,3,5)	91.08	91.98	91.12	91.08	91.03
	3	(7,6,5)	88.54	90.39	88.54	88.54	88.54
	4	(8,3,6)	89.17	90.68	89.44	89.17	89.14
	5	(7,6,7)	87.26	88.94	87.13	87.26	87.17
	AVG.		89.426	90.862	89.456	89.426	89.384
**Deit + ViT**	1	(4,3,3)	93.63	93.96	93.66	93.63	93.62
	2	(5,4,3)	92.36	92.80	92.40	92.36	92.35
	3	(3,4,3)	93.63	93.62	93.87	93.63	93.62
	4	(5,3,3)	92.99	93.52	93.04	92.99	92.99
	5	(4,3,4)	92.99	93.81	93.09	92.99	92.98
	AVG.		93.12	93.542	93.212	93.12	93.112
